# Phosphodiester backbone of the CpG motif within immunostimulatory oligodeoxynucleotides augments activation of Toll-like receptor 9

**DOI:** 10.1038/s41598-017-15178-y

**Published:** 2017-11-03

**Authors:** Jelka Pohar, Duško Lainšček, Ana Kunšek, Miša-Mojca Cajnko, Roman Jerala, Mojca Benčina

**Affiliations:** 1 Department of Synthetic Biology and Immunology, National Institute of Chemistry, Hajdrihova 19, SI-1000 Ljubljana, Slovenia; 2 Centre of Excellence EN-FIST, Trg Osvobodilne fronte 13, SI-1000 Ljubljana, Slovenia

**Keywords:** Nucleic acids, Toll-like receptors

## Abstract

Toll-like receptor 9 (TLR9) stimulatory CpG-containing oligodeoxynucleotides (ODNs) with phosphorothioate backbones have successfully replaced the naturally occurring agonists of TLR9 in drug development due to their increased stability. Replacing the nonbridging oxygen with a sulfur atom in the phosphate linkage of ODNs has been accepted as having a minor impact on the chemical and physical properties of the agonists. Here, we report that the TLR9 binding site exhibits a strong bias in favor of a phosphodiester backbone over the phosphorothioate backbone of the CpG motif. Furthermore, we show that while single point mutations of W47, W96 and K690 within the TLR9 binding site retains full TLR9 activation by phosphodiester-based ODNs, activation by phosphorothioate-based ODNs is strongly impaired. The substitution of a phosphorothioate linkage for a phosphodiester linkage of just the CpG motif considerably improves the activation potency of a phosphorothioate-based oligonucleotide for human B-cells and plasmacytoid dendritic cells, as well as for mouse bone marrow-derived dendritic cells and macrophages. Our results highlight the functional significance of the phosphodiester linkage of a CpG dinucleotide for binding, which is important in designing improved immunostimulatory TLR9 agonists.

## Introduction

Toll-like receptors (TLRs) constitute a family of pathogen recognition receptors that are activated by pathogen-associated molecular patterns of microorganisms and danger-associated molecular patterns of a host. They contribute to the induction and maintenance of innate and adaptive immunity against infectious diseases^1,2^; however, deregulation of their activity leads to the development of sepsis and autoimmune diseases^3,4^. RNA or DNA from viruses and bacteria represent natural agonists of nucleic acid-sensing TLRs. TLR3 is activated by double-stranded (ds)RNA^5,6^. The closely related TLR7 and TLR8 can be activated by single-stranded (ss)RNAs, such as exogenous viral ssRNA or endogenous RNAs released from dead cells^7,8^. In addition to longer RNA, TLR7 and TLR8 recognize degradation products of ssRNA, mono- and dinucleosides derived from the ssRNA, which are sensed through two distinct ligand-binding sites^8–10^. TLR9, however, recognizes the unmethylated cytosine-phosphate-guanine (CpG) dideoxynucleotide motif frequently present in bacteria but infrequently present in mammalian DNA. The critical role of the CpG motif in DNA structure was elucidated by Krieg *et al*.^11^. Agonistic CpG-DNA binds to TLR9 with 2:2 stoichiometry forming a symmetric TLR9-CpG-DNA complex^12^. Both protomers of the TLR9 ectodomain bind unmethylated CpG motifs of ssDNA by the N-terminal fragment (LRRNT-10) of one promoter and the C-terminal fragment (LRR20–22) of the other protomer. Unlike other TLRs, TLR9, TLR7, and TLR8 comprise a long Z-loop localized between LRR14 and LRR15 so that the proteolytic cleavage of a Z-loop is necessary for the receptor function^7,8,10^. An endosomal endonuclease DNAse II that cleaves dsDNA is crucial for the activation of TLR9 by dsDNA^13^. Moreover, dsDNA degradation with nucleases generates short oligonucleotides, which augment the activation of TLR9, as we recently demonstrated^14^.

Synthetic oligonucleotides (ODNs) with unmethylated CpG motif replace the native DNA ligands of TLR9 and are widely used to study the DNA-mediated activation of innate immunity. The activity of synthetic agonistic ODNs is affected by the position and number of CpG motifs, the sequence flanking the CpG motif, and the secondary structure formed by the ODNs^15–18^. The immunostimulatory ODNs show sequence-species specificity for the activation of TLR9^17,19^. For the activation of mouse (m)TLR9, the minimal sequence requirement of a phosphodiester (PD)-based B-type ODNs is a single CpG motif 4–6 nt separated from the 5′-end and where the 5′-TCC sequence augments activation^15^. However, the minimal ODNs that activate human (h)TLR9 comprise two CpG motifs separated by 6–10 nt where the first one is preceded by a 5′-thymidine and comprises an elongated poly-thymidine tail at the 3′-end of the ODN^16^. The substitution of three amino acid residues, Gln346, Arg348, and Gln562 of hTLR9, with mouse counterparts changes the sequence bias of hTLR9 towards the mouse less stringent sequence requirement^20^.

**Figure 1:**
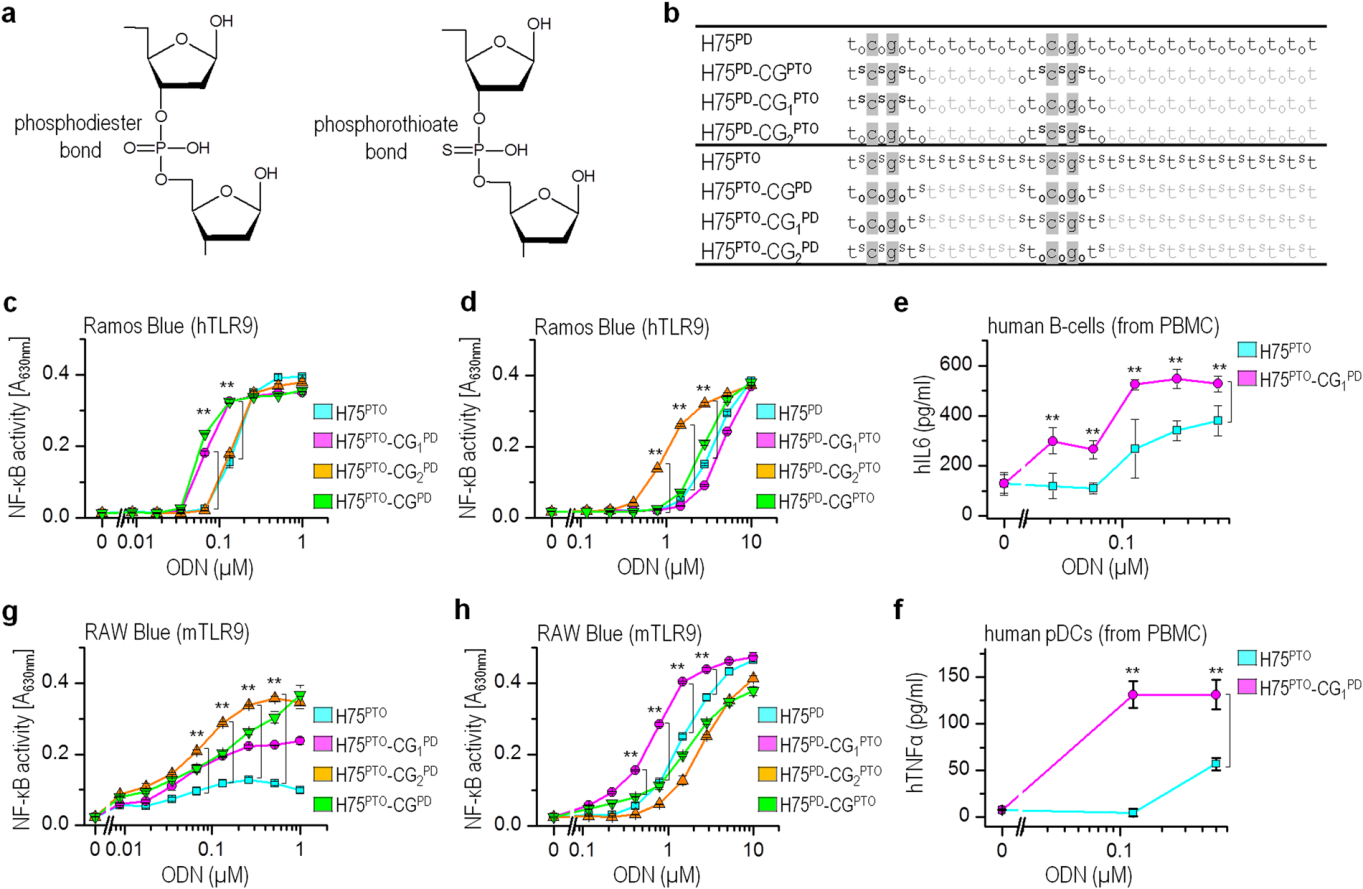
The activation efficiency of TLR9 can be tuned using ODNs with mixed backbone chemistry. (**a**) Graphics depict chemical structures of PTO and PD-based chemistries. (**b**) Schematic presentation of H75 variants. Legend: S and O depict the PTO backbone and the PD backbone, respectively; the gray shading indicates CpG motifs. (**c**,**d**) H75 with PD-based 5′-TCGT elicits the most potent activation of Ramos Blue cells. (**g**,**h**) H75 with the second PD-based CG motif triggers the best response of RAW-Blue macrophages. The Ramos Blue cells or RAW-Blue macrophages were stimulated with PD- or PTO-based H75 and their variants. The NF-κB/AP-1-regulated expression of SEAP was analyzed 18 h after stimulation. (**e**) The B-cells or (**f**) pDCs were stimulated with H75^PTO^ or its variant H75^PTO^-CG_1_^PD^. Secretion of (**e**) hIL6 (B-cells) and (**f**) hTNFα (pDCs) was analyzed 18 h after stimulation using ELISA. (*Data are representative of three*) **c**–**h**) *or two*) **e**,**f**) *independent experiments; the means of three biological replicates* ± s.d. are shown; **p < *0.05, ns p* > *0.05, unpaired two-tailed Student t-test*).

Because ODNs comprising PD backbones are degraded by nucleases, nuclease-resistant ODNs with phosphorothioate (PTO) backbones have been developed^21^. The replacement of the nonbridging oxygen with sulfur atoms (Fig. 1a) is a common step in many drug development programs. Chemical modification not only provides the nuclease resistance but also facilitates cellular uptake and bioavailability, which all enhance the activity of ODNs^11^. Therefore, synthetic ODNs may consist of a partial or a complete phosphorothioate (PTO) backbone for vaccine adjuvants and in cancer therapy^22^. However, it is not clear whether the backbone chemistry of agonistic ODNs, particularly of the CpG motif, has an immunostimulatory impact on hTLR9. Therefore, we tested the activation potency of ODNs with mixed backbone chemistry. Here, we demonstrated that a PD-based CpG motif within the PTO-based ODNs improves the activation efficiency of synthetic TLR9 agonists for human B-cells and pDCs, mouse macrophages, and mouse bone marrow-derived DCs. Using chimeric proteins between mTLR9 and hTLR9 and point mutagenesis of the selected residues within the CpG binding sites, we found that the TLR9 binding site discriminates between nonbinding oxygen and a sulfur atom in the phosphate bond of the CpG motif. Improvement in the activation efficiency of ODNs by introducing a PD backbone within the CpG motif is particularly important for the design of improved immunostimulatory synthetic hTLR9 agonists.

## Results

### Backbone of 5′CpG motif determines activation potency of ODNs for hTLR9

The substitution of nonbinding oxygen for a sulfur atom in a phosphate linkage confers a synthetic TLR9 agonist nuclease resistance and consequently improves the immunostimulatory potency of ODNs. To evaluate the impact of modified physical and chemical properties of this replacement on the activation efficiency of hTLR9, we designed synthetic agonistic ODNs with a mixed PTO and PD backbone. The minH75 (H75) was used as an ODN template and contains the minimal sequence motifs required to activate hTLR9^16^. In addition to the PD- and PTO-based H75 (H75^PD^, H75^PTO^), variants of H75 (H75 variants) were designed with mixed backbone chemistry (Fig. 1a,b; *oligonucleotide sequences can be found as* Supplementary Table S1). We substituted the first, second, or both CpG motifs with PTO-based CpG motifs within H75^PD^, generating H75^PD^-CG_1_^PTO^, H75^PD^-CG_2_^PTO^, and H75^PD^-CG^PTO^, respectively. Using a similar strategy, H75^PTO^-CG_1_^PD^, H75^PTO^-CG_2_^PD^, and H75^PTO^-CG^PD^ were designed with PTO-based CpG motifs of H75^PTO^ substituted with PD-based CpG motifs of H75^PTO^. Human B-lymphocytes (Ramos Blue cells) and human B-cells and pDCs isolated from PBMC were used to test the potency of H75 variants for the activation of TLR9. The majority of H75 variants, derived from the human-specific minH75, activated Ramos Blue cells (Fig. 1c,d); nevertheless, PTO-based H75 activated hTLR9 at approximately ten-fold lower concentrations than H75^PD^, which can be ascribed to the protective nature of the PTO backbone against the nuclease degradation^14^. To our surprise, the H75^PTO^ variants with the PD-based first CpG motif (H75^PTO^-CG_1_^PD^ and H75^PTO^-CG^PD^) were significantly more potent activators of Ramos Blue cells compared to the PTO-based first CpG motif (H75^PTO^ and H75^PTO^-CG_2_^PD^) (Fig. 1c). Similarly, the H75^PD^ variants with the PD-based first CpG were significantly better activators of hTLR9 than H75^PD^-CG_1_^PTO^ with the PTO-based first CpG motif (Fig. 1d).

The relevance of a PD-based first CpG motif for the activation of hTLR9 was also confirmed on primary cells. We isolated untouched B-cells and enriched pDCs from human PBMCs and treated the cells with H75^PTO^ and H75^PTO^-CG_1_^PD^ (Fig. 1e,f**)**. The secretion of hIL6 and hTNFα was significantly higher when B-cells and pDCs, respectively, were stimulated with H75^PTO^-CG_1_^PD^ compared to stimulation with H75^PTO^. The hIFNα expression was not affected by H75 and its variants. These data establish that the backbone chemistry of the first CpG motif of H75 contributes considerably to the activation efficiency of hTLR9.

Furthermore, we examined whether the mixed backbone chemistry of H75 shows a similar effect on the activation of mTLR9. The H75^PTO^ weakly activated RAW-Blue macrophages, as already shown by Pohar *et al*.^20^ (Fig. 1g). However, the substitution of PTO- with PD-based first, second, or both CpG motifs improved the activation efficiency of H75^PTO^ for mTLR9, with H75^PTO^-CG_2_^PD^ being the most potent activator of mTLR9. In contrast to H75^PTO^, H75^PD^ effectively activated RAW-Blue macrophages (Fig. 1h), demonstrating that the sequence-specificity for PD-based agonists is less significant for the activation of mTLR9^15^. Contrary to hTLR9, H75^PD^-CG_1_^PTO^ and not H75^PD^-CG_2_^PTO^ was the most potent activator of mTLR9 (Fig. 1g,h). These results were subsequently confirmed using mouse bone marrow-derived dendritic cells (mBM-DCs) (Supplementary Fig. S1a,b). The most potent production of mTNFα and mIL6 was triggered by the stimulation of BM-DCs with the H75^PTO^-CG_2_^PD^. Overall, for the activation of mTLR9, the second and not the first CpG motif can be used to fine-tune the potency of H75. This is not surprising, as we previously showed that the CpG motif positioned 4–6 nt from the 5′-end is important for the activation of mTLR9^15^.

Taken together, we demonstrated that the activation efficiency of H75 for hTLR9 can be significantly improved by the introduction of the PD-based first CpG motif and the PTO-based second CpG motif. To confirm that the PD substitution for the CpG motif improves the activation of other well-known B-type agonists, we selected PTO-based ODN2006 (h2006^PTO^) with four CpG motifs within ODNs. The activation of Ramos Blue cells was tested with h2006^PTO^ and its variant h2006^PTO^-CG^PD^, having substituted the PTO-based first CG dinucleotide for the PD-based first CG dinucleotide. Similar to H75, the introduced PTO-based to PD-based substitution significantly improved the activation of hTLR9 (Supplementary Fig. S1c).

### Protection of 3′ end significantly improves activation of TLR9 by PD-based ODNs

**Figure 2:**
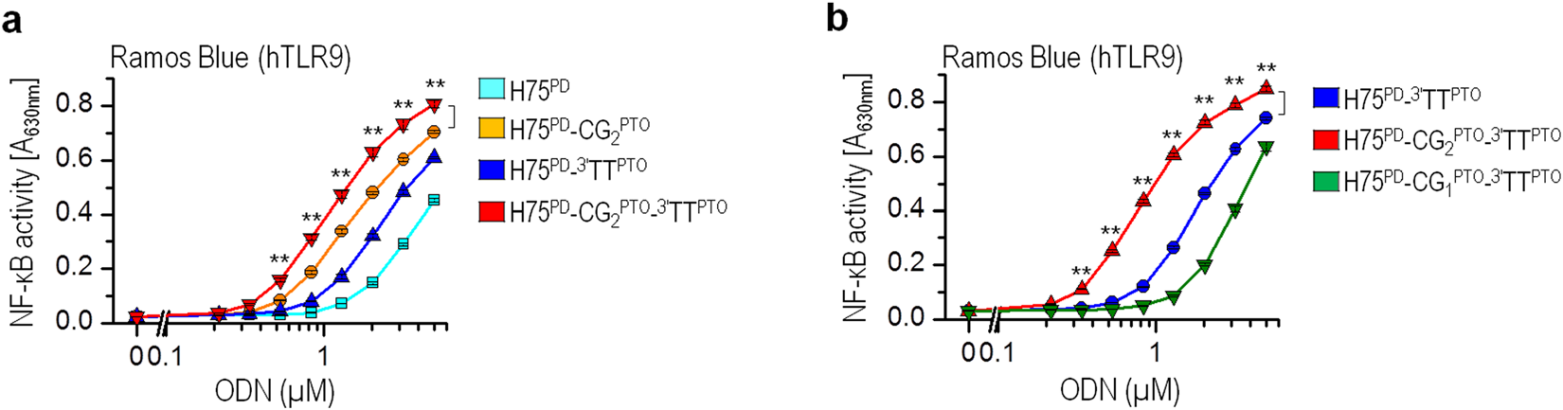
The nuclease protected 3′-end of H75 improves activation of TLR9. (**a**,**b**) The human B-lymphocytes (Ramos Blue cells) were stimulated with H75 and their variants. The NF-κB/AP-1-regulated expression of SEAP was analyzed 18 h after stimulation. (*Data are representative of three independent experiments; the means of three biological replicates* ± s.d. are shown; **p < *0.05, unpaired two-tailed Student t-test*).

Endonucleases such as DNase I and exonucleases cleave naturally occurring ssDNA and dsDNA, producing short oligonucleotides^14^ and mononucleotides; therefore, nuclease-resistant PTO-based TLR9 agonists are used in immunotherapeutic applications^21^. As the processing of exonucleases is 3′ to 5′ directional, we examined whether some protection could be achieved by modifying only the 3′-end of ODNs. To visualize the degradation dynamics and degradation products, the M80^PD^ was labeled at the 5′- and 3′-end with fluorescein isothiocyanate (FITC). In addition, an FITC label was used as a chemical modifier of thymine to protect the 3′-end against the exonucleases (Supplementary Fig. S2). M80^PD^ labeled at the 3′- end with FITC (M80^PD_^3′^FITC^) was protected from the nuclease degradation. For M80^PD_^5′^FITC^, however, degradation was already observed 4 h after incubation. No degradation of ODNs was observed in media lacking fetal bovine serum (FBS), demonstrating that the main source of nucleases is FBS. These results confirmed that the 3′-end protection of PD-based ODNs plays an important role in preventing the degradation of ODNs; therefore, it should consequently augment the activation of TLR9. An H75^PD^ variant with two 3′-end PTO-based thymines (H75s^PD^-TTT^PTO^) was prepared, where the PTO linkage should protect the ODNs against the exonucleases. These modifications improved the TLR9 activation efficiency by H75 **(**Fig. 2a). Similar to the H75 variants (Fig. 1c), the 3′-protected H75 variant with PD-based first and PTO-based second CpG motifs (H75^PD^-CG_2_^PTO^-TTT^PTO^) was the most potent activator of Ramos Blue cells (Fig. 2b). This protected variant also activated Ramos cells more effectively in comparison to the unprotected H75^PD^-CG_2_^PTO^ (Fig. 1c). However, the PTO-based first CpG motif (H75^PD^-CG_1_^PTO^-TTT^PTO^) decreased the activation potency of H75^PD^ for hTLR9 (Fig. 2b). Taken together, the 3′-end protection of H75 improves hTLR9 activation; however, the impact of first CpG motif backbone chemistry on activation efficiency remains similar to that for H75.

### The mixed backbone composition regulates potency of ODNs to activate mTLR9

**Figure 3:**
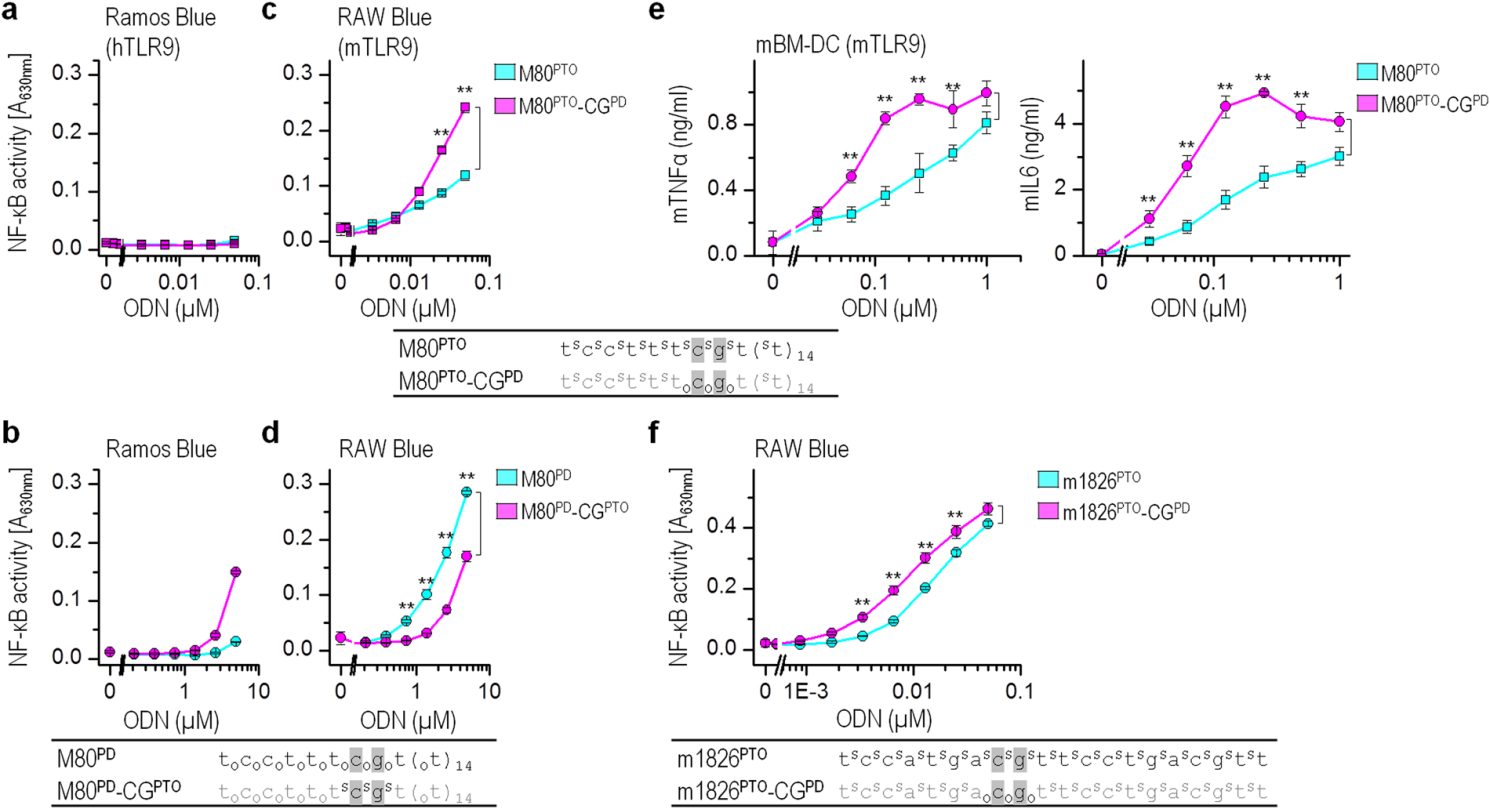
PD-based CpG motif within mouse-specific ODNs improves activation of mTLR9. The Ramos Blue cells (**a**,**b**), the RAW-Blue macrophages (**c,d,f**), or the mBM-DCs (**e**) were stimulated with PD- and PTO-based minM80 (M80), ODN1826 (m1826), and their variants. (**a–d**,**f**) The NF-κB/AP-1-regulated expression of SEAP was analyzed 18 h after stimulation. (*Data are representative of three independent experiments. Dots represent the means of three biological replicates* ± s.d.; **p < *0.05, ns p* > *0.05, unpaired two-tailed Student t-test*). (**e**) The expression of mIL6 and TNFα was analyzed 18 h after stimulation using ELISA. (*Data are representative of two independent experiments; the means of three biological replicates* ± s.d. are shown; **p < *0.05, ns p* > *0.05, unpaired two-tailed Student t-test*). Nucleotide sequences of M80 and m1826 variants. Legend: S and O depict the PTO backbone and the PD backbone, respectively; the gray shading indicates CpG motifs.

We showed that careful positioning of PTO and PD bonds in human-specific H75 significantly improves the activation of hTLR9 and mTLR9 (Fig. 1). Therefore, our next step was to verify whether the same rule applies for certain ODNs that specifically activate mTLR9. The mouse-specific minimal ODN, minM80 (M80)^15^ and ODN1826 (m1826) and its variants were tested. A single CpG motif distant from the 5′-end by 4–6 nt is sufficient to activate mTLR9, as shown for the M80^15^. The m1826, on the other hand, possesses two CG motifs positioned at a distance of 8 and 17 nt from the 5′-end. As expected, M80^PTO^ failed to activate and M80^PD^ poorly activated human B-lymphocytes (Fig. 3a,b) but effectively activated RAW macrophages (Fig. 3c,d)^15,16,20^. M80^PTO^ with the PD-based CpG motif (M80^PTO^-CG^PD^) was a more effective activator of RAW macrophages than M80^PTO^ (Fig. 3b), similar to H75^PTO^-CG_2_^PD^ activating mTLR9 (Fig. 1c). However, the PTO-based CpG motif within M80^PD^ (M80^PD^-CG^PTO^) reduced the potency to activate mTLR9 (Fig. 3d). The observed results were confirmed using mBM-DCs, with M80^PTO^-CG^PD^ significantly increasing the expression of mTNFα and mIL6 compared to M80^PTO^ (Fig. 3e).

Furthermore, we substituted the PTO-linkage of first CG dinucleotide of the ODN1826^PTO^ (m1826^PTO^) to PD-based, generating the m1826^PTO^-CG^PD^ variant. This m1826 variant activated the reporter cells better than m1826^PTO^ (Fig. 3f). The enhancement was not as strong as for the minimal ODNs (H75 and M80), which might be because the ODNs with the minimal required sequence have only the minimal required number of CpG motifs and substitution of a single motif can have a greater impact.

Taken together, the potency of PTO-based ODNs to activate TLR9 can be improved by substituting the PTO-based CpG motif backbone with the PD-based CpG motif backbone. The position of the CpG motif that backbone should be substituted from the PTO- to the PD-based differs for hTLR and mTLR9. For augmenting hTLR9 activation the 5′- CpG should be PD-based, and for mTLR9 activation the distant CpG should be PD-based. These could be due to the differences in the CpG recognition mechanism of hTLR9 and mTLR9^15,16^.

### The N-terminal region of the TLR9 ectodomain determines the sensitivity to the backbone chemistry of the first CpG motif

We showed that for the best activation of TLR9, the position of the PD-based CpG motif within the PTO-based H75 differs between hTLR9 and mTLR9 (Fig. 1c,d *vs*. 1 g,h; Fig. 1e,f *vs*. Supplementary Fig. S1a,b). These results imply that the amino acid (aa) residues of the CpG binding site of TLR9 discriminate between the nonbridging oxygen and a sulfur atom in the phosphate bond, which motivated us to determine the region of hTLR9 that senses the backbone of the CpG motif. For that purpose, we prepared the TLR9 chimeras with different substituted segments of ECD (aa 26–201, 26–427, 26–503, 26–635, and 201–635) from h to mTLR9 and *vice versa* (*detailed description of chimeras can be found as* Supplementary Table S2).

The expression and localization of chimeras transiently transfected in HEK293 cells were similar to those of the wild type (wt-)TLR9 (Supplementary Fig. S3a,b). The activation of the TLR9 chimera was tested using PTO- and PD-based mouse- and human-specific ODNs. Based on the results of the activation of the TLR9 chimera, they were classified into four groups. The chimeras h504-m-, h201-m635-h-, and m635-h- were inactive (Supplementary Fig. S4a). To rule out the functional inactivation of inactive chimeras due to folding defects and to analyze whether the loss of function affects their ability to bind ODNs, we investigated the effect of the co-expression of mutants on wt-TLR9 activation (Supplementary Fig. S4b). Inactive TLR9 chimeras that retain the ODN-binding ability should inhibit the activation of the wt-TLR9 due to the sequestration of ODNs, while folding defective mutants should have no effect. Indeed, all tested signaling-incompetent TLR9 chimeras were able to inhibit wt-TLR9 signaling. The second group comprises chimeras (h635-m-, m201-h635-m-, and m201-h-) whose response to H75 and M80 were as good or better than wt-hTLR9, regardless of the backbone chemistry of ODNs (Supplementary Fig. S4a).

**Figure 4:**
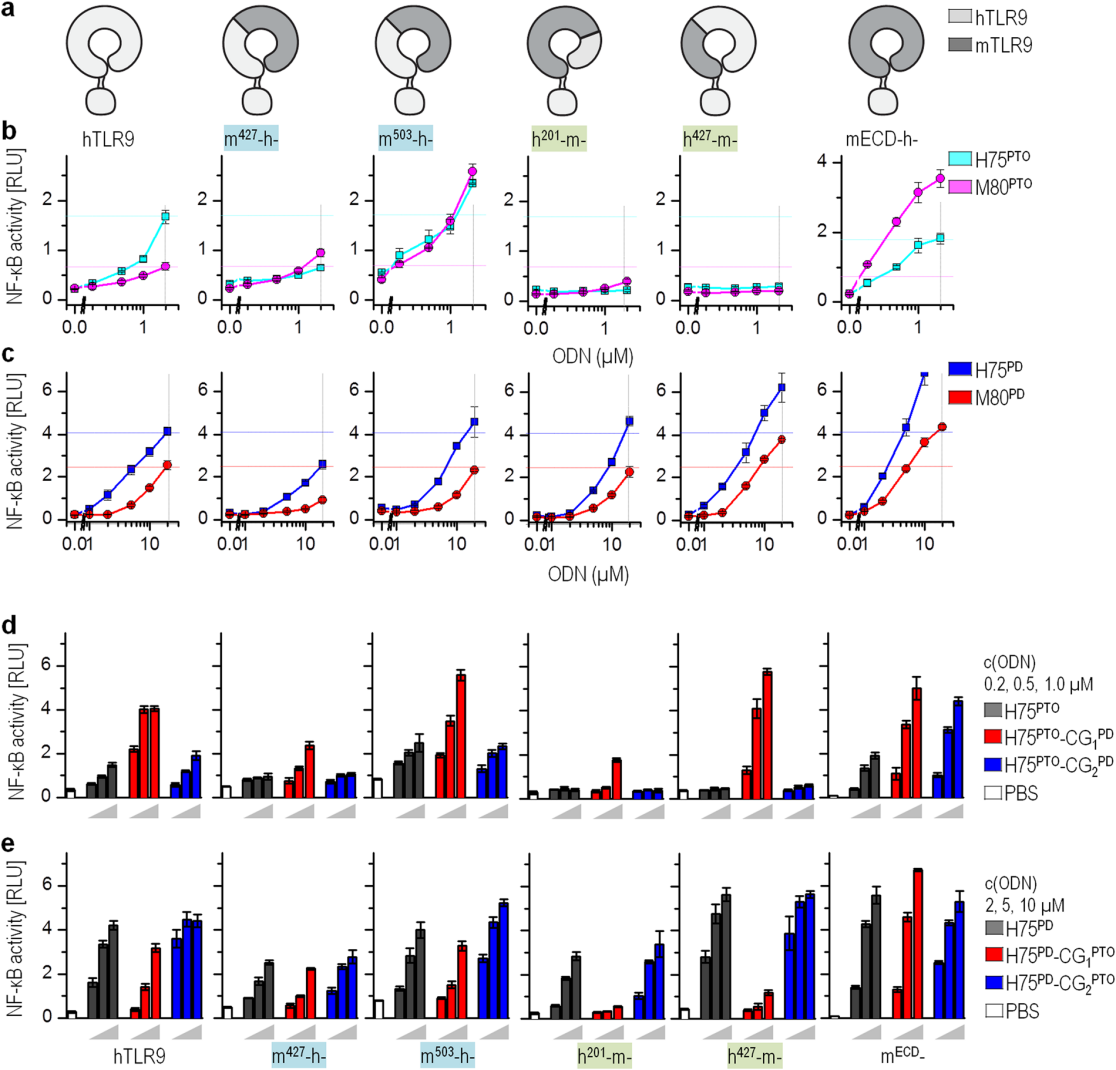
The N-terminal end of ECD defines responsiveness of TLR9 for PTO-based ODNs. (**a**) Schematic presentation of TLR9 chimera proteins. Legend: light and dark gray depicts h and mTLR9, respectively; green shading, PD-based ODNs activate the chimeras; blue shading, PTO-based ODNs activate the chimeras. (**b**–**e**) The HEK293 cells expressing wt-TLR9s and TLR9 chimeras were stimulated with PTO- or PD-based H75, M80, and H75 variants. NF-κB-dependent luciferase activity and *Renilla* luciferase activity were measured 18 h after stimulation. Relative luciferase activities are shown. (*Data are representative of two independent experiments; the means of three biological replicates* ± *s.d. are shown*). (**b**,**c**) Legend: cyan, magenta, blue, and red dotted lines show the activity of wt-hTLR9 stimulated with H75^PTO^, M80^PTO^, H75^PD^, and M80^PD^, respectively.

The third group of chimeras with the N-terminal part of hECD substituted with a mouse counterpart (m503-h-, m427-h-) could be activated with both PTO- and PD-based ODNs (Fig. 4a–c). The difference compared to wt-hTLR9 was that the chimeras were activated equally well by the PTO-based mouse- and human-specific ODNs. The wt-hTLR9 was only weakly activated by mouse-specific ODNs (M80^PTO^, m1826^PTO^)^15^. The fourth group of TLR9 chimeras with the C-terminal part of hTLR9 ECD substituted with a mouse counterpart segment (h201-m-, h427-m-) were activated by PD-based ODNs but were signaling-incompetent for PTO-based ODNs (Fig. 4a,b). The chimeras apparently retained the ability to bind PTO-based ODNs, as they reduced the activation of the co-expressed wt-TLR9 with h2006^PTO^ (Supplementary Fig. S4b). These data imply that the C-terminal segment of hECD participates in sensing and discriminating the backbone chemistry of ODNs.

To further understand how the backbone chemistry of the CpG motif affects immuno-stimulation, the sensitivity of the first CpG motif to backbone chemistry was examined for the chimeras from the third and fourth groups. As expected, the responsiveness of the third group of chimeras to the mixed backbone chemistry of H75 was similar to that for the wt-hTLR9 (Fig. 4d,e), as these chimeras responded equally well to human-specific H75^PTO^ and mouse-specific M80^PTO^. The chimeras (h201-m-, h427-m-) from the fourth group, which were signaling-incompetent to H75^PTO^, gained responsiveness to H75 with the PD-based first CpG motif (H75^PTO^-CG_1_^PD^) (Fig. 4d,e). However, the H75^PD^ with the PTO-based first CpG motif (H75^PD^-CG_1_^PTO^) failed to activate these chimeras, even though the H75^PD^ activated receptors as effectively as wt-hTLR9. These data indicate that the chimeras with the N-terminal hECD are less responsive or not responsive to ODNs with the PTO-based first CpG motif, for example, H75^PTO^, H75^PTO^-CG_2_^PD^, and H75^PD^-CG_1_^PTO^. This suggests that the properties of PD linkage within the CpG motif enable less restrictive activation of the immune response.

### Identification of the amino acid residues within the binding site that sense the backbone chemistry

Having established that the chimeras with the N-terminal segment of hECD are biased in favor of ODNs with the PD-based first CpG motif, our next step was to corroborate whether the TLR9 binding site^12^ participates in discriminating nonbinding oxygen over the sulfur atom in the phosphate backbone of agonists. Based on the structure of equine (Ec)TLR9 in combination with ODN1668_12nt^12^, we selected several aromatic and charged aa residues for point mutations (*The EcTLR9 ODN1668 _12nt structure with highlighted positions of mutated residues are shown as* Supplementary Fig. S5a). At the N-terminal site of the EcTLR9, the positive patch with K51 and R74 and aromatic residues W47, F49, and W96 were identified as important for binding the base moieties of ODN1668_12nt^12,23^ and for the formation of a binding pocket with the C-terminus of the ECD TLR9 where aa residues mainly bind the backbone of the ligand. The aromatic residues W47, F49, and W96 at the N-terminal site were changed to alanine or tyrosine (W47 only). Positively charged H641 and K690 residues at the C-terminal site proximal to the N-site were changed to the negatively charged aspartic acid. Mutation W47A but not F49A moderately affected hTLR9 activity (Supplementary Fig. S5b). However, mutation W96A and mutations H641D and K690D led to the loss of TLR9 activation when stimulated with PTO-based ODNs. The expression level and localization of mutated hTLR9s were similar to those of wt (Supplementary Fig. S5c,d). In addition, the signaling-incompetent mutants retained the ability to bind ODN^PTO^, determined through the inhibition of wt-TLR9 activation (Supplementary Fig. S5e). A binding assay revealed that the individual amino acid residue mutations are not sufficient to prevent ODN^PTO^ binding, as no difference in the binding of biotinylated h2006^PTO^ between mutants and wt-TLR9 was observed (Supplementary Fig. S5f).

**Figure 5:**
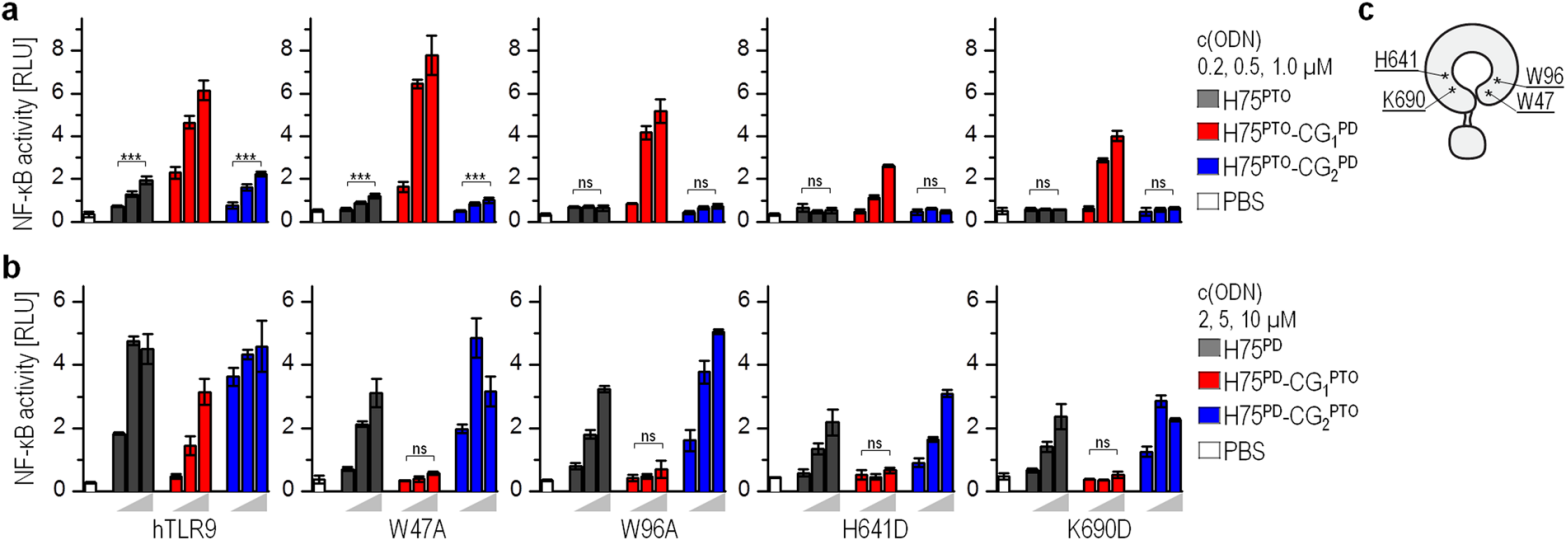
The 5′-CpG motif of minH75 is sensed by amino acid residues within the binding site. The HEK293 cells expressing wt-TLR9 and mutants were stimulated with (**a**) minH75^PTO^ and its variants or (**b**) minH75^PD^ and its variants. (**c**) Scheme of hTLR9 with the position of amino acid residues selected for mutation. NF-κB-dependent luciferase activity and *Renilla* luciferase activity were measured 18 h after stimulation. Relative activities after the subtraction of the intrinsic activity of non-stimulated cells are shown. (*Data are representative of two independent experiments; the means of three biological replicates* ± s.d. are shown; **p < *0.05, ns p* > *0.05, unpaired two-tailed Student t-test*).

All four selected mutants of hTLR9 when stimulated with H75^PTO^, and its variants with the PTO-based first CpG motif (H75^PTO^-CG_2_^PD^ and H75^PD^-CG_1_^PTO^) failed to activate an NF-κB response (Fig. 5a,b). However, the substitution of the PTO-based backbone of the first CpG motif of H75^PTO^ with the PD-based backbone (H75^PTO^-CG_1_^PD^) restored the activation potency of ODNs for the mutants (Fig. 5a). Furthermore, ODNs with the PD-based first CpG motif (H75^PD^, H75^PD^-CG_2_^PTO^, and H75^PTO^-CG_1_^PD^) (Fig. 5b) activated mutants as effectively as wt-hTLR9. Overall, these data establish that the amino acid residues W47, W96, H641, and K690 of hTLR9 that correspond to the amino acid residues of the CpG binding site of EcTLR9 discriminate between the naturally occurring PD linkage of the CpG dinucleotide and the synthetic PTO linkage of the CpG dinucleotide.

## Discussion

Our research focuses on evaluating the impact of a nonbinding atom in a phosphate bond of the CpG motif on the immunostimulatory potential of synthetic TLR9 agonists. We demonstrated that the backbone chemistry of the CpG dinucleotide of ODNs significantly affects TLR9 activation. Our results highlight that the CpG binding site of TLR9 distinguishes between the nonbridging oxygen and a sulfur atom of the CpG phosphate linkage, with a bias in favor of the oxygen atom. First, we found that the substitution of PTO with the PD-based 5′-end CpG motif significantly improved the hTLR9 activation efficiency of PTO-based human-specific ODNs. Furthermore, the PD-based CpG motif 6 nt separated from the 5′-end of mouse-specific ODN^PTO^ improved the activation of mTLR9. With chimeric proteins between m- and hTLR9 and site-directed mutagenesis, we found that the N- and C-terminal parts of ECD forming a CpG-binding site sense the backbone chemistry. These data identify nonbridging oxygen of a phosphate bond within the CpG motif as superior to a sulfur atom and demonstrate that an improvement in ODN activation potency can be achieved with carefully positioned PD and PTO backbones within synthetic TLR9 agonists.

The replacement of nonbinding oxygen for a sulfur atom in a phosphate linkage renders oligonucleotides particularly nuclease-resistant^24^. Therefore, this conserved type of modification has been rapidly accepted in therapeutic applications due to the markedly enhanced immunostimulatory power^21^. However, the replacement of nonbridging oxygen by a sulfur atom changes the physical and chemical properties of the phosphate bond: increased Van der Waals radius (1.85 Å P = S vs. 1.44 Å P = O) with lengthening of the P-S bond (2.0 Å P = S vs. 1.5 Å P = O) and a changed partial charge distribution of the phosphate moiety^25,26^. This replacement also creates a chiral center (“Sp” or “Rp” conformation) at each phosphate bond, which generates multiple isomers of the oligo. These individual isomers have been shown to have different physical and chemical properties^27^; therefore, it is not unexpected that the receptor response should be different. However, it is somewhat surprising that the effects of replacing PD with PTO are so localized. While our study highlights that the backbone chemistry of the CpG motif affects agonist potency to activate TLR9, it also raises the question of what the molecular mechanism of TLR9 is behind the favoring of nonbridging oxygen over sulfur in the phosphate linkage within particular CpG motifs. Based on the crystal structures of the EcTLR9 ectodomain in combination with 12 nt ODN1668 ligand^12^, the base moieties of the CpG motif are recognized by the lateral face of the N-terminus from LRRNT to LRR2, and LRR21 and LRR22 interact with the backbone of the CpG motif with the direct involvement of H641, H642, F667, and N694. Mutations of W47 and W96 at the N-terminus and of H641 and K690 at C-terminus resulted in reduced or abolished activation in response to PTO-based agonists. However, the ODNs with PD-based 5′-TCG (first CpG motif) effectively activated the hTLR9 mutants, indicating that phosphate bond chemistry significantly effects the binding of the unmethylated CpG motif to TLR9. We propose that smaller sized PD bond and partial charge distribution leading to a more positive phosphate atom and an overall increase in the negative charge density of other oxygen atoms^26^ will make the interaction of a CpG dinucleotide with aa residues of the CpG binding pocket stronger and therefore less sensitive to point mutations within TLR9. It is difficult to completely exclude the impact of PTO bond chirality on the CpG motif– TLR9 binding mechanism, although it is interesting that a change as small as the replacement of a PTO linkage of a single CpG motif with a PD linkage of a single CpG motif significantly improves the activation potency of PTO-based ODNs.

Although the PD-based CpG motif within PTO-based ODNs improved the activation of TLR9, the optimal position of the PD-based CpG motif differs for h and mTLR9. We propose that the observed species-specific CpG position preferences originate from the fact that the sequence preferences of hTLR9 differ from those of mTLR9. It has become clear in recent years that the recognition of synthetic agonists by TLR9 is sequence- and species-dependent^11,15,16,18,20,28–30^. We demonstrated previously that 5′-TCG trinucleotide is critical for the activation of hTLR9^16^. However, the CpG motif distant from the 5′-end critically affects the activation of mTLR9^15^. A mixed PTO-PD backbone sequence is characteristic for the A-class ODNs. The central PD-based palindrome with a single CpG motif is critical for TLR9 activation and the PTO-based poly G-tail at the 5′- and 3′-ends protects the ODN against exonuclease activity. For activation of TLR9, the A-class ODNs must first be cut with DNase II in endolysosomes^13^, where 11–12-mer fragments are generated. The backbone structure of the active 11-mer fragment resembles B-class ODNs with the PD-based TCG motif at the 5′-end and a PTO-based 3′-tail.

Replacing nonbridging oxygens with sulfur atoms changes the key properties of ODNs, making them more suitable for cell biology research applications and therapeutics. The PTO bonds integrated near the 5′- and 3′- ends protect the ODNs against exonucleases, and their incorporation throughout the ODN sequence protects them against the endonucleases but with some undesired consequences. PTO-modified ODNs trigger a strong multi-factor activation of nucleophiles and their adherence to collagen-coated surfaces irrespective of CpG content^31^. Moreover, PTO ODNs bind to platelets, leading to strong platelet CpG-independent activation^32^, and induce a CpG-independent respiratory burst, while PD ODNs do not^33^. However, this may be due to the much longer lifetime of PTO ODNs in the circulation. The binding site of the unmethylated CpG motif of TLR9 has been perfected during evolution to strongly detect naturally occurring agonists, making it insensitive to the effects of some point mutations. Therefore, PD-based synthetic agonists, particularly PD-based CG dinucleotides, might significantly improve the TLR9 activation efficiency of PTO-based ODNs.

Analyzing the effect of replacing PD to PTO linkage for the PD-based ODNs, the results are even more surprising. A phosphate linkage replacement of the 5′-end CpG motif with PTO, as expected, reduced the activation potency of the ODNs, while a PTO substitution of CpG motif distant from the 5′-end improved the activation of ODNs compared to PD-based ODNs with or without 3′-end protection. We propose that the CpG motif with PTO linkage is less favored for TLR9 binding, which results in binding only the 5′-end PD-based CpG motif and in improving TLR9 activation. It is important to realize that the PTO bond modification should not be incorporated at every available position in ODNs. For the optimal physiological effect, it should rather be introduced sparingly and with careful consideration of the impact each bond might have.

## Materials and Methods

### Cell lines

Mouse leukemic monocyte macrophages (RAW-Blue macrophages derived from RAW264.7, which expressed a NF-κB/AP-1-inducible secreted embryonic alkaline phosphate (SEAP) reporter gene; InvivoGen) and human embryonic kidney cell lines (HEK293; ATCC CRL-1573 and HEK293T; ATCC CRL-3216) were cultured in DMEM media with 1 g/l glucose, 2 mM L-glutamine, 10% (v/v) heat-inactivated fetal bovine serum (FBS) (Invitrogen) at 37 °C in 5% CO_2_. B-lymphocytes (Ramos-Blue cells, which stably expressed an NF-κB/AP-1-inducible SEAP reporter gene; InvivoGen) were cultured in Iscove’s Modified Dulbecco’s Medium (IMDM, Invitrogen) with 10% (v/v) heat-inactivated FBS at 37 °C in 5% CO_2_.

### Mouse bone marrow-derived dendritic cells, mBM-DCs, and cytokine detection

Mouse bone marrow (mBM) was obtained by flushing the femurs and tibiae taken from 8–10-week-old wt C57BL/6 OlaHsd mice with RPMI-1640 and 2% (v/v) heat-inactivated FBS. After the lysis of the red blood cells (RBCs) with RBC lysis buffer (BioLegend) for 5 min on ice, the cells (1 × 10^6^ cells/ml) were cultured at 37 °C for a week in media containing RPMI-1640, 10% (v/v) heat-inactivated FBS, 2 mM L-glutamine, penicillin (100 U/ml), streptomycin (0.1 mg/ml), IL4 (5 ng/ml), mGMC-SF (10 ng/ml) (for BM-derived dendritic cells (BM-DC)^GMC-SF,IL4^), and 50 mM 2-mercaptoethanol. At day 3, an equal volume of fresh media was added to the cells. At day 6, immature mBM-DCs were harvested. The cells were seeded onto 96-well round-bottom plates (Corning) (1 × 10^5^ cells/100 µl/well) and stimulated with ODNs (concentrations indicated on graphs). The mIL6 and mTNFα secreted in the media were determined using ELISA (mIL6 or mTNFα ELISA Ready-Set-Go, eBioscience). Error bars represent the s.d. values obtained from at least three biological replicates.

All appropriate measures were taken to minimize pain and discomfort in experimental animals collecting the femurs and tibiae. All of the procedures involving animals were performed according to the directives of the EU 2010/63 and were approved by the Administration of the Republic of Slovenia for Food Safety, Veterinary, and Plant Protection of the Ministry of Agriculture, Forestry, and Foods, Republic of Slovenia (Permit no. U34401-37/2015/5).

### Human plasmacytoid dendritic cells, pDCs, and B-cells isolated from peripheral blood mononuclear cells (PBMCs)

The study was approved by the local Medical Ethics Committees (No. 142/10/13) and all methods were carried out in accordance with the relevant local regulatory requirements. A written informed consent was obtained from the blood donors. Human PBMCs were extracted from whole blood using the density gradient medium Lymphoprep and SepMate tubes (StemCell Technologies). Human plasmacytoid dendritic cells (pDCs) were isolated from PBMCs by the depletion of non-target cells using Plasmacytoid Dendritic Cell Isolation Kit II (Miltenyi Biotec, cat. no. 130-097-415). The LD column and QuadroMACS magnet were used for separation. The cells were seeded onto 96-well round-bottom plates (Corning) (0.25 × 10^5^ cells/100 µl/well) in media containing RPMI1640, 10% (v/v) heat-inactivated FBS, 2 mM L-glutamine, penicillin (100 U/ml), streptomycin (0.1 mg/ml), and 50 mM 2-mercaptoethanol and were stimulated with ODNs (concentrations indicated on the graphs) for 20 h. The secreted hTNFα was determined using ELISA (human TNF alpha Ready-Set-Go ELISA, eBioscience).

Untouched B-cells were isolated from PBMCs by negative selection with a CD43 MicroBeads Kit (Miltenyi Biotec, cat. no. 130-091-333). The LS column and QuadroMACS magnet were used for separation. The cells were seeded onto 96-well round-bottom plates (Corning) (2.5 × 10^5^ cells/100 µl/well) in media containing RPMI-1640, 10% (v/v) heat-inactivated FBS, 2 mM L-glutamine, penicillin (100 U/ml), streptomycin (0.1 mg/ml), and 50 mM 2-mercaptoethanol and were stimulated with ODNs (concentrations indicated on the graphs) for 20 h. The hIL6 secreted in the media was determined using ELISA (hIL-6 ELISA Ready-Set-Go, eBioscience). Error bars represent the s.d. values obtained from at least three biological replicates.

### Plasmids and reagents

Expression plasmids containing a human (h)TLR9 gene (pUNO-hTLR9^HA^) and mouse (m)TLR9 (pUNO-mTLR9^HA^) were obtained from InvivoGen, and TLR9^YFP^ (pcDNA3-TLR9-YFP) was obtained from Addgene. The plasmid phRL-TK constitutively expressing *Renilla* luciferase for the normalization of transfection efficiency was obtained from Promega. Plasmid coding for firefly luciferase under the NF-κB promoter (pELAM-1-luciferase) was a gift from C. Kirschning (Institute for Medical Microbiology, University of Duisburg-Essen, Essen, Germany). For site-directed mutagenesis, C-terminal HA-tagged hTLR9 (hTLR9^HA^) was used. The chimeras between hTLR9 and mTLR9, inserted into the pUNO vector, are depicted in Supplementary Table S1. Cells were treated with different TLR9 agonists (Supplementary Table S2 depicts nucleotide sequences): human-specific ODN2006 (h2006) and minH75 (H75)^16^ and mouse-specific ODN1826 (m1826) and minM80 (M80)^15^ (all B-class ODNs), with a PTO backbone, a PD backbone, or a mixed PTO–PD backbone (Integrated DNA Technologies). The 3′- and 5′-end FITC labeled M80 were obtained from Integrated DNA Technologies.

### Reporter assays

HEK293 cells plated at 2.2 × 10^4^ cells/well (0.1 ml; White 96-well plates) were transiently transfected with plasmids expressing wild type (wt-)TLR9, mutants or chimeras (20 ng DNA/well), UNC93B1 (5 ng DNA/well), ELAM1-luciferase reporter plasmid (50 ng DNA/well), and phRL-TK (5 ng DNA/well) (unless stated otherwise) using Lipofectamine 2000 (Invitrogen) or polyethylenimine (PEI) (ratio 8:1 PEI (µl):DNA (µg)). The total amount of DNA for each transfection was kept constant by adding pcDNA3 plasmid (Invitrogen). After 24 h, the culture medium was replaced with fresh medium, and the cells were stimulated with TLR9 agonists for 18 h.

To analyze whether the loss-of-function mutations maintained their ability to bind ligands, we tested the effect of the co-expression of mutants or chimeras (4 and 10 ng DNA/well) and TLR9^HA^ (2 ng DNA/well) on NF-κB activation upon stimulation with ODNs. After cell lysis in a passive lysis buffer (Promega), reporter activities were analyzed using a dual-luciferase reporter assay. Relative luciferase activity (RLU) was calculated by normalizing each sample’s firefly luciferase activity with the constitutive *Renilla* luciferase activity determined in the same sample. Relative luciferase activity (NF-kB activity) was calculated after the subtraction of intrinsic activity (RLU of cells treated with buffer). Each experiment was repeated independently at least two times and performed in at least three biological parallels (mean ± s.d.). A Student’s unpaired two tailed t-test was used for statistical comparison.

Ramos Blue or RAW-Blue macrophages were seeded at a density of 2 × 10^5^ cells/well (IMDM with 10% FBS) or 0.5 × 10^5^ cells/well (DMEM with 4.5 g/L glucose with 10% FBS) (0.1 ml; 96-well plates), respectively. The cells were immediately stimulated with ODNs. After 20 h, the supernatants were collected and the NF-κB/AP-1 activation linked to SEAP was determined using a Quanti Blue reagent (Invitrogen). The means with s.d. of three biological replicates are given. The data are representative of at least two independent experiments.

### Immunoblotting

HEK293T cells were seeded at 2.2 × 10^5^ cells/well (1 ml, 12-well plate). After 24 h, the cells were transiently transfected with plasmids expressing TLR9s^HA^ (1470 ng DNA/well), UNC93B1 (5 ng), and plasmid constitutively  expressing mCerulean (10 ng, for transfection control) using Lipofectamine 2000 transfection reagent. Forty-eight hours later, the cells were lysed using a RIPA buffer (50 mM Tris pH 7.5, 150 mM NaCl, 1% (v/v) Triton X-100, 0.1% (w/v) SDS, 0.5% (w/v) deoxycholate) with complete mini protease inhibitors (Roche Applied Science). Proteins from the supernatant were separated by SDS-PAGE and transferred to a Hybond ECL nitrocellulose membrane (GE Healthcare). The membrane was washed and incubated in a blocking buffer (1 × PBS, 0.1% (w/v) Tween20, 0.2% (w/v) I-Block (Tropix)). Blots were incubated with primary antibodies: rabbit anti-HA (1:1000; H6908, Sigma), rabbit anti-GFP (1:1000; A11122, Invitrogen), or mouse anti-β-actin (1:1000; 3700, Cell Signaling), followed by incubation with secondary goat anti-mouse IgG-HRP (1:4000; sc-2005, Santa Cruz) antibodies and goat anti-rabbit IgG-HRP (1:4000; ab6721, Abcam) and detection with the ECL Western blotting detection reagent (GE Healthcare).

### Confocal imaging analysis

HEK293T cells seeded at 3.5 × 10^5^ cells/well (8-well culture chamber; Ibidi) were co-transfected with the plasmids expressing the TLR9^HA^ mutant or chimera (140 ng DNA/well) and the wt-hTLR9^YFP^ (80 ng DNA/well). After 48 h, the TLR9^HA^ was stained using rabbit anti-HA (1:50; H6908, Sigma) and secondary goat anti-rabbit IgG conjugated with AlexaFluor 647 (2 mg/ml; A21246, Invitrogen). Successive images of wt-hTLR9^YFP^ and TLR9^HA^ mutants or chimeras excited at 514 nm (fluorescence emission 525–570 nm) and 633 nm (fluorescence emission 650–700 nm), respectively, were captured. Images were acquired using a Leica TCS SP5 inverted laser-scanning microscope on a Leica DMI 6000 CS module equipped with an HCX Plane-Apochromat lambda blue 63 × oil-immersion objective with NA 1.4 (Leica Microsystems). Images were processed with LAS AF software (Leica Microsystems) and ImageJ software (National Institute of Mental Health, Bethesda, Maryland, USA).

### Detection of degradation of ODNs

For the visualization of the degradation of ODNs, the M80^PD^ was labeled at the 5′- or 3′-end with FITC (IDT Technologies). FITC labeled ODNs (M80^PD_^3′^FITC^, M80^PD_^5′^FITC^) were added to growth media with or without FBS (final concentration, 1 µM). Samples were collected 4 h and 21 h later. An equal amount (4 µl, which equals 4 pmol of ODNs) of growth media with labeled M80 or the size standard (GeneRuler Ultra Low Range DNA Ladder; Thermo Scientific) were mixed with 1.25 × loading buffer (95% formamide, 15 mM EDTA), heated (5 min at 95 °C), and separated using 20% TBE-urea polyacrylamide gel in TBE buffer at 200 V for 20 min. The FITC-labeled M80 was imaged using IVIS Lumina (PerkinElmer). Subsequently, the gels were stained for 20 min with SYBR Gold Nucleic Acid Gel Stain (Invitrogen), diluted in TBE buffer (1:10000), and imaged using IVIS Lumina (to detect the standard).

### AlphaScreen TLR9 binding assay

The AlphaScreen assay is a bead-based nonradioactive binding assay system used to detect biomolecular interactions. The binding of the biological partners brings the donor and acceptor beads into close proximity, and AlphaScreen acceptor beads emit a broad signal between 520 and 620 nm. The assays were performed in a final reaction volume of 20 µl of the assay buffer, which contained 50 mM MOPS-NaOH (pH 6.5), 100 mM NaCl, 0.01% (w/v) Tween-20, and 1 g/l BSA in a ProxiPlate 384 (PerkinElmer). The h2006^PTO^ labeled with biotin-tetraethyleneglycerol (TEG) at 3′ (h2006^PTO^-b) (Sigma) was used. In the assay, 4 µl cell lysate (prepared as described below) was incubated with an h2006^PTO^-b (up to 75 µM final concentration) for 1 h at 23 °C. Anti-HA acceptor beads (20 µg/ml f.c.) were added and incubated for 1 h at 30 °C. Next, streptavidin-coated donor beads (20 µg/ml f.c.) were added and incubated for 30 min at 30 °C before the signals were measured with EnVision (PerkinElmer). The binding of h2006^PTO^-b to wt and mutant TLR9 after the subtraction of intrinsic activity (no h2006^PTO^-b) are shown as AlphaScreen units (all n = 3, ± s.d.; two independent experiments).

HEK293T cells seeded onto a 24-well plate (Techno Plastic Products) at 1.5 × 10^5^ cells/well were transiently transfected with phRL-TK plasmid expressing *Renilla* luciferase as a transfection control (10 µg DNA/well), UNC93B1 (5 ng DNA/well), and plasmid expressing hTLR9^HA^ wt or mutants, or pcDNA3 plasmid (control) (735 µg DNA/well) using Lipofectamine 2000 transfection reagent (Invitrogen). After 48 h, cells were lysed in 0.2 ml lysis buffer (50 mM Tris-HCl (pH 7.4), 1 mM EDTA, 150 mM NaCl, 0.5% Triton X-100, 20% glycerol, 1 mM Na_3_VO_4_, 25 mM NaF, and 1 mM PMSF) containing a complete mini protease inhibitor (Roche) for 10 min on ice. Next, cell lysates were centrifuged (10,000 rpm, 10 min, 4 °C). The transfection efficiency was determined based on *Renilla* luciferase activity. In AlphaScreen assays, the amounts of cell extracts containing equal *Renilla* luciferase activity were used.

### Software and statistics

Graphs were prepared using Origin 8.1 (http://www.originlab.com/), and GraphPad Prism 5 (http://www.graphpad.com/) was used for statistics. A Student’s unpaired two-tailed t-test (equal variance was assessed with the F-test, assuming normal data distribution) or an analysis of variance (ANOVA) test followed by a Tukey’s post hoc test were used for statistical comparison of data. For all tests, p values less than 0.05 were considered statistically significant.

### Availability of data and material

The data that support the findings of this study are disclosed in the paper and its supplementary information file. The raw data are available from the authors upon reasonable request and with permission of the National Institute of Chemistry.

## Electronic supplementary material

Supplementary Information
