# Publisher Correction: Phosphodiester backbone of the CpG motif within immunostimulatory oligodeoxynucleotides augments activation of Toll-like receptor 9

**DOI:** 10.1038/s41598-017-17988-6

**Published:** 2018-01-05

**Authors:** Jelka Pohar, Duško Lainšček, Ana Kunšek, Miša-Mojca Cajnko, Roman Jerala, Mojca Benčina

**Affiliations:** 1 Department of Synthetic Biology and Immunology, National Institute of Chemistry, Hajdrihova 19, SI-1000 Ljubljana, Slovenia; 2 Centre of Excellence EN-FIST, Trg Osvobodilne fronte 13, SI-1000 Ljubljana, Slovenia

*Scientific Reports* **7**:14598; doi:10.1038/s41598-017-15178-y; Article published online 03 November 2017

The original HTML version of this Article contained a typographical error in the publication date ‘03 November 2017’ which was incorrectly given as ‘06 November 2017’. This has now been corrected in the HTML version of the Article.

