# Publisher Correction: Phosphodiester backbone of the CpG motif within immunostimulatory oligodeoxynucleotides augments activation of Toll-like receptor 9

**DOI:** 10.1038/s41598-018-22376-9

**Published:** 2018-03-06

**Authors:** Jelka Pohar, Duško Lainšček, Ana Kunšek, Miša-Mojca Cajnko, Roman Jerala, Mojca Benčina

**Affiliations:** 1 Department of Synthetic Biology and Immunology, National Institute of Chemistry, Hajdrihova 19, SI-1000 Ljubljana, Slovenia; 2 Centre of Excellence EN-FIST, Trg Osvobodilne fronte 13, SI-1000 Ljubljana, Slovenia

Correction to: *Scientific Reports* 10.1038/s41598-017-15178-y, published online 03 November 2017

An error occurred after publication resulting in the inadvertent omission of the Article and Volume numbers from the PDF version of this Article. This error has now been corrected in the PDF version of the Article; the HTML version was correct from the time of publication.

