# Supplementary material for: Phosphodiester backbone of the CpG motif within immunostimulatory oligodeoxynucleotides augments activation of Toll-like receptor 9
Source: Sci Rep. 2017 Nov 3;7:14598. doi: 10.1038/s41598-017-15178-y (PMC5668283; doi:10.1038/s41598-017-15178-y)
Supplement: Supplementary file 1 — Supplementary Information [file 41598_2017_15178_MOESM1_ESM.pdf]

Phosphodiester backbone of the CpG motif within immunostimulatory oligodeoxynucleotides augments activation of Toll-like receptor 9

Jelka Pohar,[1] Duško Lainšček,[1] Ana Kunšek,[1] Miša-Mojca Cajnko,[1] Roman Jerala,[1,2,*] and Mojca Benčina[1,*]

[1] Department of Synthetic Biology and Immunology, National Institute of Chemistry, Hajdrihova 19, SI-1000 Ljubljana, Slovenia

[2] Centre of Excellence EN-FIST, Trg Osvobodilne fronte 13, SI-1000 Ljubljana, Slovenia

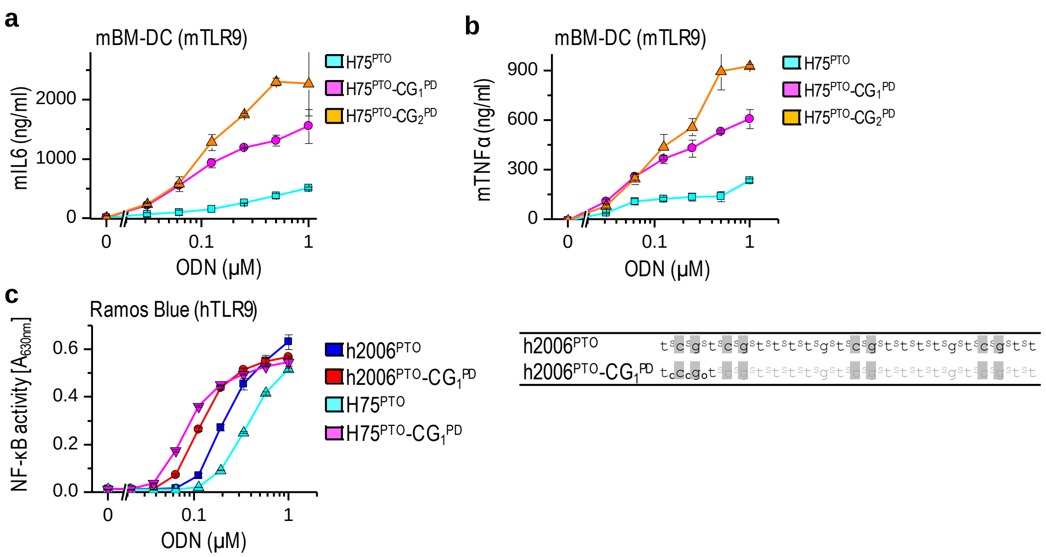

Supplementary Fig. S1. (**a**,**b**) **The second CpG motif of H75 fine-tunes the response of mBM-DCs.** The expression of (**a**) mIL6 and (**b**) TNFα was analyzed using ELISA 18 h after the stimulation of mBM-DCs with $H75^{PTO}$ and its variants. (**c**) **The PD-based first CpG motif improves the activation efficiency of ODN2006.** The Ramos Blue cells were stimulated with $H75^{PTO}$, $h2006^{PTO}$, and their variants. The NF-κB/AP-1-regulated expression of SEAP was analyzed 18 h after stimulation. Schematic presentation of ODN2006 variants. Legend: S and O depict the PTO backbone and the PD backbone, respectively; the gray shading indicates CpG motifs. (*Data are representative of two (**a**,**b**) or three (**c**) independent experiments; the means of three biological replicates ± s.d. are shown; **p<0.05, ns p>0.05, unpaired two-tailed Student t-test*).

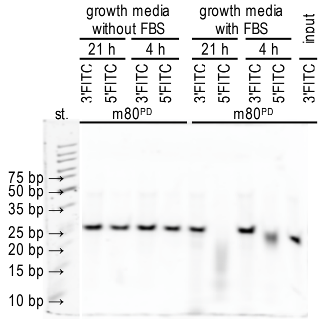

Supplementary Fig. S2. Degradation of 3’-end unprotected ODNs is facilitated by FBS. PD-based 3’- or 5’-end FITC labeled M80 (M80$^{PD_3'FITC}$, M80$^{PD_5'FITC}$) were added to growth media without or with FBS. Samples were collected 4 h and 21 h after stimulation. Fluorescently labeled oligonucleotides were separated by 20% TBE-urea polyacrylamide gel electrophoresis and visualized using IVIS Lumina.

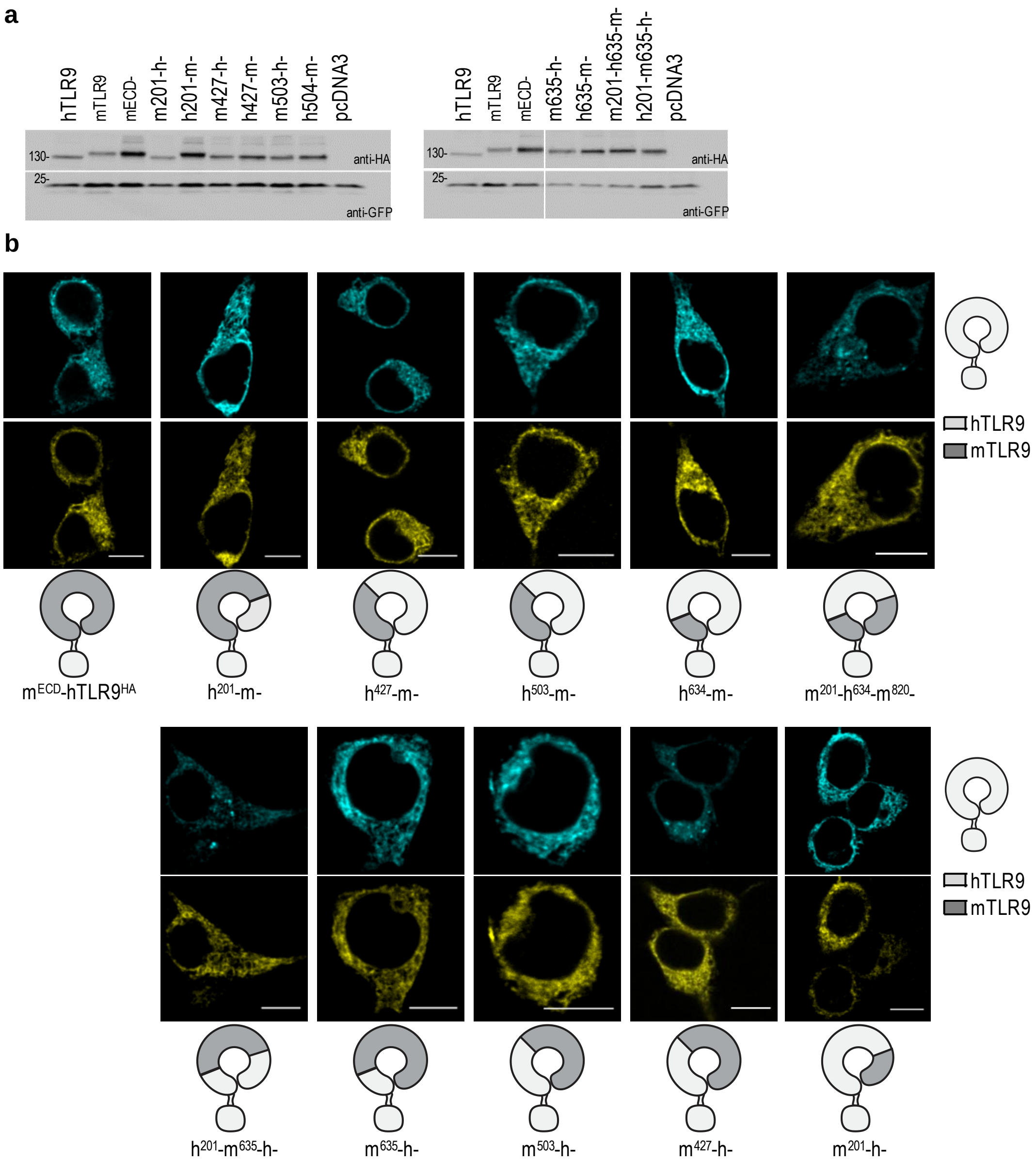

Supplementary Fig. S3. (**a**) Expression and (**b**) localization of the TLR9 chimeras equal those of wt-TLR9. (A) The expression of wt-TLR9^{HA} and chimeras determined by Western blot analysis using anti-HA antibodies. Loading control β-actin and transfection control GFP were detected with anti-GFP antibodies. (*Data are representative of two independent experiments*.) (**b**) The localization of the wt-hTLR9^{YFP} (cyan) and the HA-tagged chimeric TLR9 (yellow) was analyzed 48 h after the transfection of HEK293 cells. TLR9 chimeras were detected by staining cells with anti-HA primary antibodies and secondary goat anti-rabbit IgG conjugated with AlexaFluor 647. (*Data are representative of two independent experiments*.) Legend: light and dark gray depicts h and mTLR9.

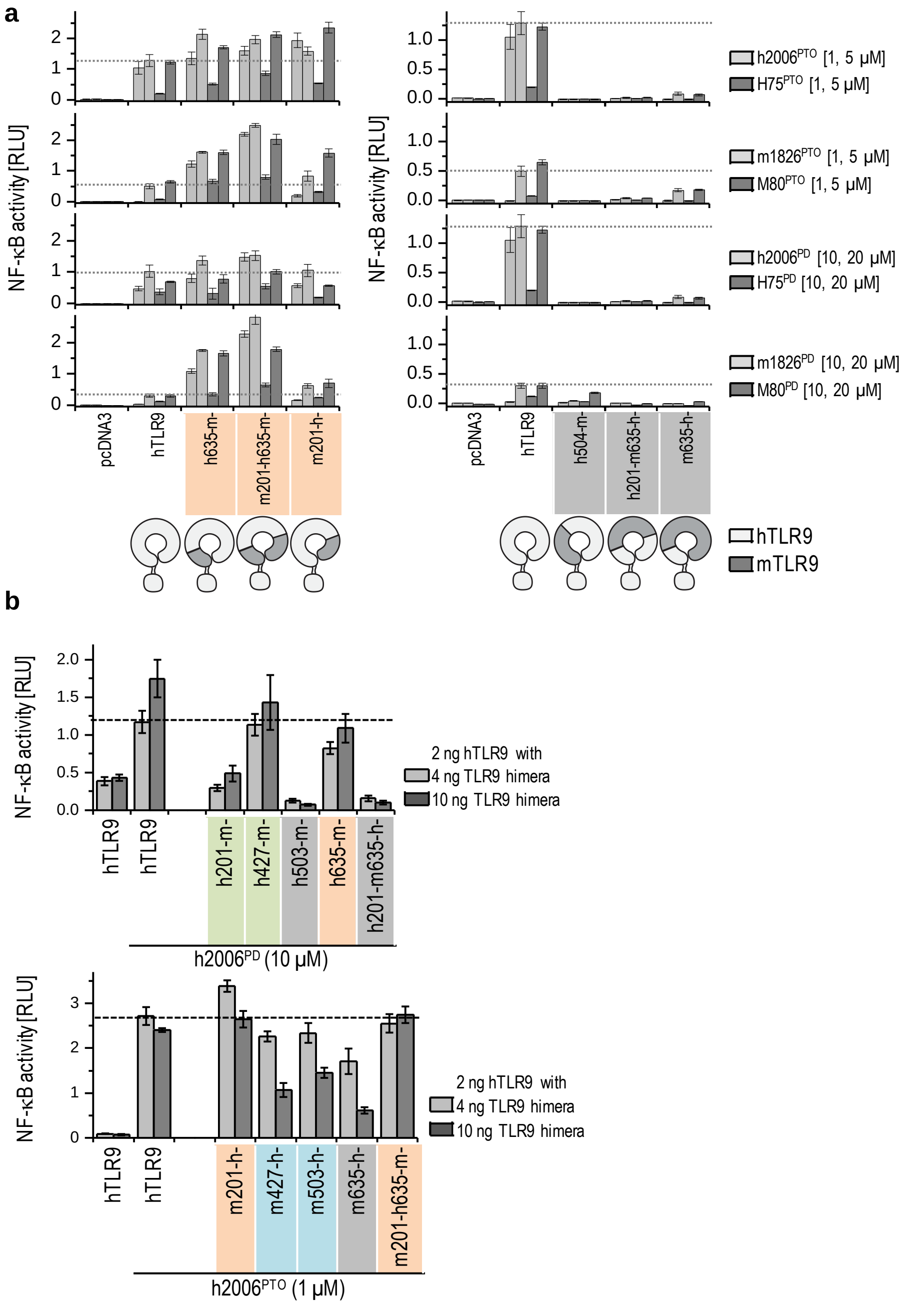

Supplementary Fig. S4. (**a**) **The TLR9 chimeras are classified into four groups with respect to activation profiles.** HEK293 cells expressing wt-TLR9s and TLR9 chimeras were stimulated with the PTO- or PD-based human- or mouse-specific minimal ODNs (H75, h2006, M80, and m1826). (**b**) **When co-expressed with wt-hTLR9, the inactive TLR9 chimera inhibited TLR9 activity.** HEK293 cells co-transfected with plasmids expressing wt and chimera $TLR9^{HA}$ were stimulated with $hODN2006^{PD}$ or $hODN2006^{PTO}$. NF-κB-dependent luciferase activity and *Renilla* luciferase activity were measured 18 h after stimulation. Relative activities after the subtraction of the intrinsic activity of non-stimulated cells are shown. (*Data are representative of two independent experiments; the means of four biological replicates ± s.d. are shown*). Legend: orange shading, TLR9 chimeras behave similar to wt-hTLR9; gray, inactive TLR9 chimera; green, PD-based ODNs activate the chimeras; blue, PTO-based ODNs activate the chimeras.

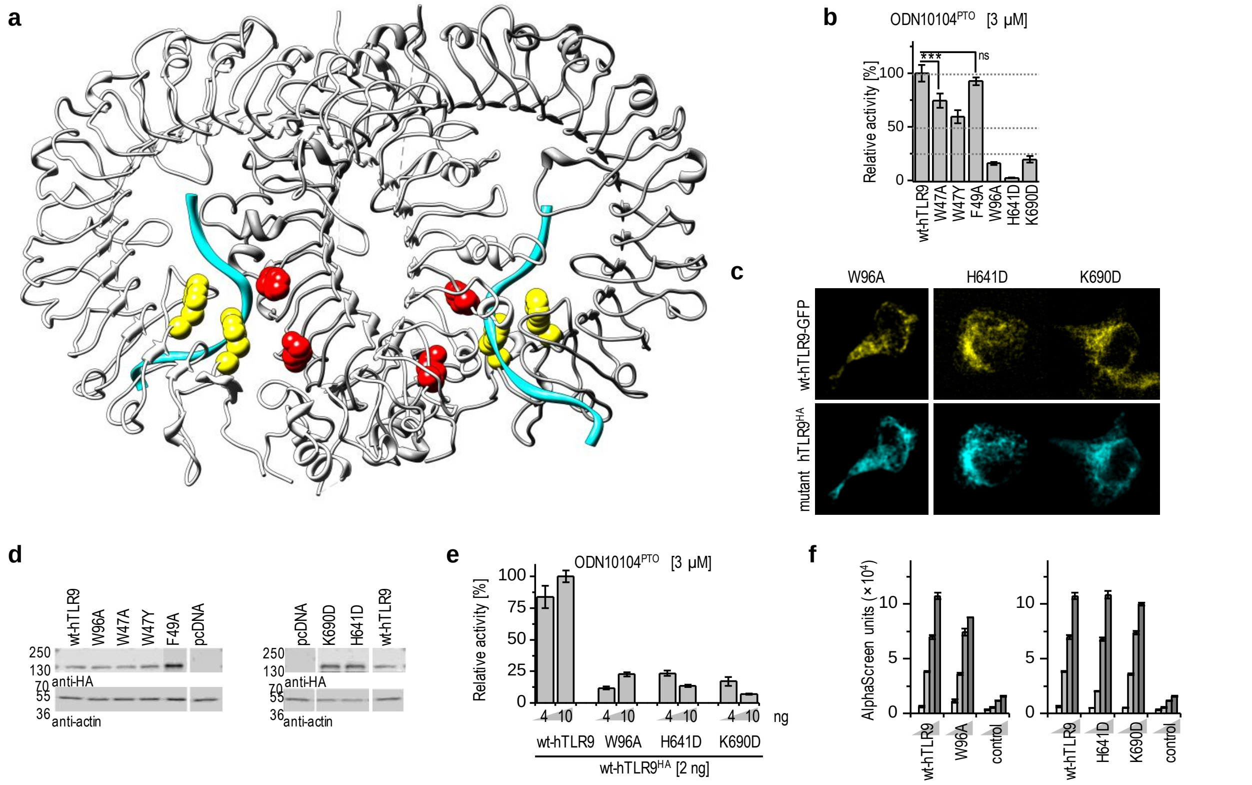

Supplementary Fig. S5. Selected mutations of hTLR9 do not impair ODN binding, expression, and localization of TLR9. (**a**) The EcTLR9:ODN1668_12nt structure with highlighted mutated residues. Legend: grey, TLR5 ectodomain; cyan, ODN1668_12nt; yellow, W47 and W96; red, H641 and K690. (**b**) Mutations of W96 at site N of one protomer and H641and K690 at site C of the other protomer render the TLR9 inactive. HEK293 cells transfected with plasmid-expressing wt-hTLR9 or mutants were stimulated with hODN10104^{PTO}. NF-κB-dependent luciferase activity was measured. Relative activities of mutants compared to wt-TLR9 were calculated after subtracting the intrinsic activity for each ODN. NF-κB-dependent luciferase activity and *Renilla* luciferase activity were measured 18 h after stimulation. Relative activities after the subtraction of the intrinsic activity of non-stimulated cells are shown. (*Data are representative of two independent experiments; the means of three biological replicates* ± *s.d. are shown; **$p<0.05$, ns $p>0.05$, unpaired two-tailed Student t-test*). (**c**) Expression and (**d**) localization of hTLR9 mutants. (**c**) Microscopic images of hTLR9^{HA} mutants (cyan) and wt-hTLR9^{YFP} (yellow) are depicted. (**d**) Western blot analysis of wt TLR9^{HA} and mutants. (**e**) TLR9 mutants when co-expressed with wt-hTLR9 inhibited TLR9 activity. HEK293 cells co-transfected with plasmids expressing wt and mutant TLR9^{HA} were stimulated with ODN10104^{PTO}, and NF-κB dependent luciferase activity was measured. Relative activity of mutants compared to wt was calculated after subtracting the intrinsic activity. (*Data are representative of two independent experiments; the means of three biological replicates* ± *s.d. are shown*). (**f**) hTLR9^{HA} mutants bind h2006^{PTO}-b (20–60 µM) similarly to wt-hTLR9^{HA}. The AlphaScreen method with streptavidin donor and anti-HA acceptor beads was used to measure ODN binding to TLR9. The control refers to cell extract obtained from HEK293 cells transfected with pcDNA3 plasmid. (*The means of three biological replicates* ±*s.d. are shown*.)

Supplementary Table S1. List of TLR9 wild type and TLR9 chimera proteins.

Name:	Composition:
$hTLR9^{HA}$	h^{26-818}- hTM-TIR
$mTLR9^{HA}$	m^{26-820}- mTM-TIR
mECD-	m^{26-820}- hTM-TIR
h^{201}-m-	h^{26-201}-$m^{202-820}$- hTM-TIR
h^{418}-m-	h^{26-418}-$m^{419-820}$- hTM-TIR
h^{427}-m-	h^{26-427}-$m^{428-820}$- hTM-TIR
h^{504}-m-	h^{26-504}-$m^{503-820}$- hTM-TIR
h^{635}-m-	h^{26-635}-$m^{634-820}$- hTM-TIR
m^{201}-h^{635}-m-	m^{26-201}-$h^{202-635}$-$m^{635-820}$- hTM-TIR
m^{201}-h-	m^{26-201}-$h^{202-818}$- hTM-TIR
m^{418}-h-	m^{26-418}-$h^{419-818}$- hTM-TIR
m^{427}-h-	m^{26-427}-$h^{428-818}$- hTM-TIR
m^{503}-h-	m^{26-503}-$h^{503-818}$- hTM-TIR
m^{635}-h-	m^{26-635}-$h^{634-818}$- hTM-TIR
h^{201}-m^{634}-h-	h^{26-201}-$m^{202-634}$-$h^{636-818}$- hTM-TIR

Legend: h = human; m = mouse; HA = Hemaglutinin tag; TM = transmembrane segment; TIR = The Toll/interleukin-1 receptor (TIR) domain

Supplementary Table S2. List of oligonucleotides (ODNs)

Name:	Sequence (5’-3’):
H75^{PD}	$t_oc_og_ot_ot_ot_ot_ot_ot_ot_oc_og_ot_ot_ot_ot_ot_ot_ot_ot_ot_ot_ot$
H75^{PD}-CG^{PTO}	$t^sc^sg^st_ot_ot_ot_ot_ot_ot^sc^sg^st_ot_ot_ot_ot_ot_ot_ot_ot_ot_ot_ot$
H75^{PD}-CG$_1^{PTO}$	$t^sc^sg^st_ot_ot_ot_ot_ot_ot_oc_og_ot_ot_ot_ot_ot_ot_ot_ot_ot_ot_ot$
H75^{PD}-CG$_2^{PTO}$	$t_oc_og_ot_ot_ot_ot_ot_ot^sc^sg^st_ot_ot_ot_ot_ot_ot_ot_ot_ot_ot_ot$
H75^{PD}-3’TT^{PTO}	$t_oc_og_ot_ot_ot_ot_ot_ot_oc_og_ot_ot_ot_ot_ot_ot_ot_ot_ot^st^st$
H75^{PD}-CG$_1^{PTO}$-3’TT^{PTO}	$t^sc^sg^st_ot_ot_ot_ot_ot_ot_oc_og_ot_ot_ot_ot_ot_ot_ot_ot_ot^st^st$
H75^{PD}-CG$_2^{PTO}$-3’TT^{PTO}	$t_oc_og_ot_ot_ot_ot_ot_ot^sc^sg^st_ot_ot_ot_ot_ot_ot_ot_ot_ot^st^st$
H75^{PTO}	$t^sc^sg^st^st^st^st^st^st^st^sc^sg^st^st^st^st^st^st^st^st^st^st^st$
H75^{PTO}-CG^{PD}	$t_oc_og_ot^st^st^st^st^st^st_oc_og_ot^st^st^st^st^st^st^st^st^st^st^st$
H75^{PTO}-CG$_1^{PD}$	$t_oc_og_ot^st^st^st^st^st^st^st^sc^sg^st^st^st^st^st^st^st^st^st^st^st$
H75^{PTO}-CG$_2^{PD}$	$t^sc^sg^st^st^st^st^st^st^st_oc_og_ot^st^st^st^st^st^st^st^st^st^st^st$
M80^{PD}	$t_oc_oc_ot_ot_ot_oc_og_ot_ot_ot_ot_ot_ot_ot_ot_ot_ot_ot_ot_ot_ot_o$
M80^{PD}-CG^{PTO}	$t_oc_oc_ot_ot_ot^sc^sg^st_ot_ot_ot_ot_ot_ot_ot_ot_ot_ot_ot_ot_ot_ot_o$
M80^{PTO}	$t^sc^sc^st^st^st^sc^sg^st^st^st^st^st^st^st^st^st^st^st^st^st^st^st_o$
M80^{PTO}-CG^{PD}	$t^sc^sc^st^st^st_oc_og_ot^st^st^st^st^st^st^st^st^st^st^st^st^st^st^st$
M80$^{PD\text{-}5’FITC}$	FITC-$t_oc_oc_ot_ot_ot_oc_og_ot_ot_ot_ot_ot_ot_ot_ot_ot_ot_ot_ot_ot_ot$
M80$^{PD\text{-}3’FITC}$	$t_oc_oc_ot_ot_ot_oc_og_ot_ot_ot_ot_ot_ot_ot_ot_ot_ot_ot_ot_ot_ot_ot_o$-FITC
h2006^{PTO}	$t^sc^sg^st^sc^sg^st^st^st^st^sg^st^sc^sg^st^st^st^st^sg^st^sc^sg^st^st$
h2006^{PTO}-CG^{PD}	$t_oc_og_ot^sc^sg^st^st^st^st^sg^st^sc^sg^st^st^st^st^sg^st^sc^sg^st^st$
m1826^{PTO}	$t^sc^sc^sa^st^sg^sa^sc^sg^st^st^sc^sc^st^sg^sa^sc^sg^st^st$
m1826^{PTO}-CG^{PD}	$t^sc^sc^sa^st^sg^sa_oc_og_ot^st^sc^sc^st^sg^sa^sc^sg^st^st$

Legend: ^{S} = phosphorothioate linkage; $_O$ = phosphodiester linkage; abbreviations H75, M80, h2006, and m1826 stand for minH75, minM80, ODN2006, and ODN1826, respectively. CG motifs are marked with grey shading. FITC = fluorescein isothiocyanate.
